# Supplementary material for: Fatal amyloid formation in a patient’s antibody light chain is caused by a single point mutation
Source: eLife. 2020 Mar 10;9:e52300. doi: 10.7554/eLife.52300 (PMC7064341; doi:10.7554/eLife.52300)
Supplement: Figure 2—source data 1. [file elife-52300-fig2-data1.docx]

**Figure 2_source data 1.** Crystallographic data collection and refinement statistics.

|  | **WT-1** | **Pat-1** |
| --- | --- | --- |
|  |  |  |
| **Crystal parameters** |  |  |
| Space group | P6_3_22 | P6_1_22 |
| Cell constants | a = b = 104.5 Å  c = 55.2 Å | a = b = 127.3 Å  c= 81.8 Å |
| Subunits / AU^a^ | 1 | 2 |
|  |  |  |
| **Data collection** |  |  |
| Beam line | ID-30, ESRF | ID-30, ESRF |
| Wavelength (Å) | 0.976 | 0.976 |
| Resolution range (Å)^b^ | 50 – 1.55 (1.65 – 1.55) | 50 – 2.5 (2.6 – 2.5) |
| No. observed reflections | 162247 | 115993 |
| No. unique reflections^c^ | 26220 | 13924 |
| Completeness (%)^b^ | 99.7 (99.8) | 99.4 (100) |
| R_merge_ (%)^b, d^ | 4.3 (58.7) | 5.5 (49.6) |
| I/σ (I)^b^ | 20.9 (3.2) | 20.6 (4.2) |
|  |  |  |
| **Refinement (REFMAC5)** |  |  |
| Resolution range (Å) | 15 – 1.55 | 15 – 2.5 |
| No. refl. working set | 24867 | 13148 |
| No. refl. test set | 1309 | 692 |
| No. non hydrogen | 1025 | 1653 |
| Solvent (H_2_O, ions, PEG) | 178 | 39 |
| R_work_ / R_free_ (%)^e^ | 14.2 / 16.5 | 16.2 / 22.7 |
| r.m.s.d. bond (Å) / (°)^f^ | 0.007 / 1.3 | 0.003 / 1.2 |
| Average B-factor (Å^2^) | 25.4 | 83.6 |
| Ramachandran Plot (%)^g^ | 95.4 / 4.6 / 0 | 95.3 / 4.7 / 0 |
|  |  |  |
| PDB accession code | 6SM1 | 6SM2 |

^[a]^ Asymmetric unit

^[b]^ The values in parentheses for resolution range, completeness, R_merge_ and I/σ (I) correspond to the highest resolution shell

^[c]^ Data reduction was carried out with XDS and from a single crystal.

^[d]^ R_merge_(I) = Σ_hkl_Σ_j_ | I(hkl)_j_ - <I(hkl)> | / Σ_hkl_ Σ_j_ I(hkl)_j_, where I(hkl)_j_ is the j^th^ measurement of the intensity of reflection hkl and <I(hkl)> is the average intensity

^[e]^ R = Σ_hkl_ | |F_obs_| - |F_calc_| |/Σ_hkl_ |F_obs_|, where R_free_ is calculated without a sigma cut off for a randomly chosen 5% of reflections, which were not used for structure refinement, and R_work_ is calculated for the remaining reflections

^[f]^ Deviations from ideal bond lengths/angles

^[g]^ Percentage of residues in favored / allowed / outlier region
