# Supplementary material for: Fatal amyloid formation in a patient’s antibody light chain is caused by a single point mutation
Source: eLife. 2020 Mar 10;9:e52300. doi: 10.7554/eLife.52300 (PMC7064341; doi:10.7554/eLife.52300)
Supplement: Figure 6—source data 1. [file elife-52300-fig6-data1.docx]

**Figure 6_source data 1.** Hydrophobic interaction pattern of the three mutated amino acids at positions 15, 81 and 82, located in the altered hydrophobic surface area of the V_L_ variants Pat-1 and WT-1.

| **Pat-1** | FR2 | | FR3 | | | | FR4/C-terminus | | |
| --- | --- | --- | --- | --- | --- | --- | --- | --- | --- |
|  | **L15** | **I19** | **I78** | **V81** | **L82** | **A83** | **V109** | **V111** | **L112** |
| **L15** |  |  |  |  |  |  |  |  |  |
| **V81** |  |  |  |  |  |  |  |  |  |
| **L82** |  |  |  |  |  |  |  |  |  |
|  |  |  |  |  |  |  |  |  |  |
| **WT-1** | FR2 | | FR3 | | | | FR4/C-terminus | | |
|  | **P15** | **I19** | **I78** | **L81** | **Q82** | **A83** | **V109** | **V111** | **L112** |
| **P15** |  |  |  |  |  |  |  |  |  |
| **L81** |  |  |  |  |  |  |  |  |  |
| **Q82** |  |  | R64 | E84 | D85 | E86 |  |  |  |
